# Designing the best breeding strategy for *Coffea canephora*: Genetic evaluation of pure and hybrid individuals aiming to select for productivity and disease resistance traits

**DOI:** 10.1371/journal.pone.0260997

**Published:** 2021-12-29

**Authors:** Emilly Ruas Alkimim, Eveline Teixeira Caixeta, Tiago Vieira Sousa, Itamara Bomfim Gois, Felipe Lopes da Silva, Ney Sussumu Sakiyama, Laércio Zambolim, Rodrigo Silva Alves, Marcos Deon Vilela de Resende

**Affiliations:** 1 Federal University of Triângulo Mineiro, Iturama, MG, Brazil; 2 Brazilian Agricultural Research Corporation—Embrapa Café, Viçosa, MG, Brazil; 3 Federal Institute of Triângulo Mineiro, Campina Verde, MG, Brazil; 4 Federal University of Viçosa, Viçosa, MG, Brazil; 5 National Institute of Coffee Science and Technology—INCT Café, Lavras, MG, Brazil; Swedish University of Agricultural Sciences, SWEDEN

## Abstract

Breeding programs of the species *Coffea canephora* rely heavily on the significant genetic variability between and within its two varietal groups (conilon and robusta). The use of hybrid families and individuals has been less common. The objectives of this study were to evaluate parents and families from the populations of conilon, robusta, and its hybrids and to define the best breeding and selection strategies for productivity and disease resistance traits. As such, 71 conilon clones, 56 robusta clones, and 20 hybrid families were evaluated over several years for the following traits: vegetative vigor, incidence of rust and cercosporiosis, fruit ripening time, fruit size, plant height, canopy diameter, and yield per plant. Components of variance and genetic parameters were estimated via residual maximum likelihood (REML) and genotypic values were predicted via best linear unbiased prediction (BLUP). Genetic variability among parents (clones) and hybrid families was detected for most of the evaluated traits. The Mulamba-Rank index suggests potential gains up to 17% for the genotypic aggregate of traits in the hybrid population. An intrapopulation recurrent selection within the hybrid population would be the best breeding strategy because the genetic variability, narrow and broad senses heritabilities and selective accuracies for important traits were maximized in the crossed population. Besides, such strategy is simple, low cost and quicker than the concurrent reciprocal recurrent selection in the two parental populations, and this maximizes the genetic gain for unit of time.

## Introduction

The *Coffea* genus has great economic and social importance, with Brazil being the world’s largest producer and exporter [1]. Within the *Coffea* genus, the three most important commercial species are *Coffea arabica*, *Coffea canephora*, and *Coffea liberica* [2]. In Brazil, the cultivation of *C*. *arabica* expanded rapidly, mainly due to the superior quality of the beverage. However, between the years 1870 and 1900, there was a high incidence of rust caused by the fungus *Hemileia vastatrix* in *C*. *arabica* crops. As such, the species *C*. *canephora* began to be included in breeding programs as it presents resistance to the pathogen [3], along with greater rusticity, high productive potential, and high levels of total soluble solids [4].

*C*. *canephora* stands out as it has two varietal groups with extensive genetic variability that is used in breeding programs [5]. The varietal groups conilon (*Coffea canephora* var. Conilon) and robusta (*Coffea canephora* var. Robusta) have distinct and complementary characteristics that are important also for the exploitation of heterosis in breeding programs [6]. Conilon coffee trees are more tolerant to drought and constitute the main varieties developed in Brazil, while robusta coffee trees are resistant to coffee rust [7].

The current taxonomy system for the species *C*. *canephora* splits it into two major groups of germplasm: the Congolese and the Guineans. The Congolese group is divided in the SG1 (which encompasses the “*C*. *canephora* var. Conilon”, planted in Brazil) and SG2 (which encompasses the “*C*. *canephora* var. Robusta”, planted worldwide) subgroups. SG1 and SG2 are compounded by many populations. In the past, the Guineans used to be called Kouilou (originating from Ivory Coast). Nowadays, the Congolese SG1 subgroup clusters the varieties named Kouilou originated from Benin and Gabon and corresponds to the cultivated Brazilian conilon. The Congolese SG2 subgroup comprehends all the varieties so called robusta in the past, originated from Central Africa [8]. The term conilon derives from the name Kouilou, which is the name of a coastal river flowing into the Atlantic Ocean in southern Gabon. SG1 subgroup is known as conilon coffee and the others are known as robusta coffee [8].

Molecular and phenotypic differences do exist between conilon and robusta types. Provided that there are dominance effects on the targeted traits, such divergences are likely to promote heterosis and hybrid vigour in the crossed population. Then, some breeding strategies aiming at improving hybrid populations between conilon and robusta types are justified.

In *C*. *canephora*, especially in Brazil, there is a lack of comparative studies involving alternative breeding strategies stemming from intrapopulation and interpopulation improvement programs in this species [8, 9]. According to Montagnon et al. [8], only Ivory Coast could implement a Reciprocal Recurrent Selection (RRS) program, as only this country has the whole genetic diversity available in its field collections. This RRS strategy improves the heterosis of the hybrid interpopulation and is well adapted to hybrid seed varietal release, more suitable than clonal cuttings for a massive varietal diffusion. Apart from Ivory Coast, countries are often relying on clonal mass selection from recombining populations [9].

According to Ferrão et al. [9] and Leroy et al. [10], results of the studies in Ivory Coast showed the presence of genotypic and phenotypic variability within and between the Guinean and Congolese populations for plant architecture, resistance to drought, vegetative vigor, organoleptic characteristics, resistance to pests and diseases; superiority of the hybrids descended from the intergroup cross in relation to the intragroup cross, justifying the efficiency of RRS; efficiency of recurrent selection in populations breeding per se and in obtaining genetically superior hybrids; and variation from 0.22 to 0.78 of heritabilities for traits related to plant architecture, from 0.13 to 0.40 for vegetative vigor and from 0.15 to 0.28 for those associated with beans weight. The phenotypic and genotypic correlations between plant productivity and vegetative vigor were high; there was a low correlation of bean yield with vegetative vigor and production; there was a high correlation between the crown diameter of the four-year-old plants with the productivity of two to five-year-old plants [9].

Regarding the estimates of genetic gains for different traits, Montagnon et al. [8] and Leroy et al. [10] verified significant progress, whose productivity of hybrids involving elite parents from the both populations were from 16% to 140% higher than the two commercial clonal varieties, and the most productive cross was more vigorous. By selecting 5% of the best plants, they showed high genetic gain expectations with a value higher than 60% for production; moderated with rates of 14% to 18% for young plant vegetative vigor and low for canopy diameter, being possible to predict 60% of the genetic gains in selected plant productivity compared to the most productive clone used as control. Taken together, improving interpopulation hybrids seems promising and studies on comparative hybrid populations strategies are needed and should be prioritized.

The objectives of this study were to evaluate parents and families from the populations of conilon, robusta, and its hybrids and to define the best breeding and selection strategies for productivity and disease resistance traits.

## Materials and methods

### Genetic materials and experimental design

The studied conilon coffee population consisted of 71 clones (S1 Table in S2 File) that were evaluated in two trials, each with 34 clones and three controls (UFV 3629–11, UFV 3628–2, and UFV 513). The robusta coffee population consisted of 56 clones (S2 Table in S2 File) evaluated in two trials, each with 27 clones and two controls (UFV 3366–134 and UFV 3366–139). Each experimental trial used a randomized block design with five replications and one plant per plot.

The hybrid coffee population consisted of 20 families obtained by controlled crossbreeding between five conilon coffee clones (male genitors) and five robusta coffee clones (female genitors) (S3 Table in S2 File). The hybrids were evaluated using a randomized block design with 1 to 35 (average number 11.90) repetitions and one plant per plot, obtained from seeds.

Planting was done at a spacing of 3.0 m between rows and 1.5 m between plants. The conilon and robusta clones were planted in July 2009 and the hybrids were planted in March 2011. Throughout the experimental period, the plants received the treatments necessary for cultivation based on Matiello et al. [11], but without pruning or the use of chemicals to control diseases.

### Phenotyping

Phenotyping was carried out at the time of fruit physiological maturity for seven consecutive years (2010 to 2016) in the conilon and robusta coffee populations and for five consecutive years (2012 to 2016) in the hybrid population. The following traits were assessed: vegetative vigor, incidence of rust and cercosporiosis, time of fruit ripening, fruit size, plant height, canopy diameter, and yield per plant.

The vegetative vigor was assessed using a scale from 1 to 10, where a score of 1 was assigned to highly depleted plants and 10 was assigned to highly vigorous plants. The incidence of rust (caused by the fungus *Hemileia vastatrix* Berk. & Br.) and cercosporiosis (caused by the fungus *Cercospora coffeicola* Berk. & Cooke) were evaluated on a scale of 1 to 5, where 1 was assigned to asymptomatic plants and 5 was assigned to plants highly susceptible to the pathogen. The fruit ripening time was classified as early, medium or late, with scores of 1 to 3, respectively. Fruit size was classified as small, medium or large, with scores of 1 to 3, respectively. Plant height (cm) was obtained by measuring from ground level to the apical point of the most developed orthotropic branch. Canopy diameter (cm) was measured as the canopy projection perpendicular to the planting row. Yield per plant was obtained by measuring the total volume of fruits, in liters.

### Statistical analyses

Coffee trees have a long reproductive cycle, with fluctuations in production over time (biennial), and differences in precocity and productive longevity [12]. For this reason, accurate, efficient, and informative statistical methods, such as the residual maximum likelihood/best linear unbiased prediction (REML/BLUP) procedure, have been recommended for the genetic analysis of coffee trees [13, 14], considering phenotypic [14–21] and genomic [22–26] data.

There are several advantages in using the REML/BLUP procedure as it enables the incorporation of kinship information, comparisons of genotypes over time and space, correction of environmental effects, as well as the simultaneous estimate of variance components and prediction of genetic values. Moreover, it deals well with complex data structures and can be applied to unbalanced data and non-orthogonal designs [27, 28]. Then, this statistical approach is especially suited to coffee breeding.

Variance components and genetic parameters were estimated via REML [29] and genotypic values were predicted via BLUP [30]. All statistical analyses were performed using the Selegen-REML/BLUP software [31].

#### Conilon and robusta coffee populations

To evaluate the conilon and robusta coffee clones, the following linear mixed model was used [32]:

y=Xu+Zg+Wb+Tp+e;

where, y is the vector of phenotypic data; u is the vector of year or crop effects (assumed as fixed), added to the general average; g is the vector of genotypic effects (assumed as random); b is the vector of block effects across experiments (coded sequentially and assumed as random); p is the vector of permanent environmental effects (assumed as random); and e is the vector of residuals (random). The capital letters (X, Z, W, and T) represent the incidence matrices of these effects.

The average and variance structures associated with this model are [33]: $y|u,\;V∼N(Xu,\;V);\;g|\sigma_{g}^{2}∼N(0,\;I\sigma_{g}^{2});\;b|\sigma_{b}^{2}∼N(0,\;I\sigma_{b}^{2});\;p|\sigma_{p}^{2}∼N(0,\;I\sigma_{p}^{2})$; and $e|\sigma_{e}^{2}∼N(0,\;I\sigma_{e}^{2})$; where, V is the phenotypic variance and equals $ZZ^{\prime }\sigma_{g}^{2}+WW^{\prime }\sigma_{b}^{2}+TT\prime \sigma_{p}^{2}+I\sigma_{e}^{2}$; I is an identity matrix; and $\sigma_{g}^{2},\;\sigma_{b}^{2},\;\sigma_{p}^{2}$ and $\sigma_{e}^{2}$ are the genotypic variances among progenies, blocks, permanent environment, and residuals, respectively. The covariances between these effects are equal to zero.

The mixed model equations associated with this model are [33]:

$$[\begin{array}{c} \hat{u}\\ \hat{g}\\ \hat{b}\\ \hat{p} \end{array}]={\left[\begin{array}{cccc} X^{\prime }X & X^{\prime }Z & X^{\prime }W & X^{\prime }V\\ Z^{\prime }X & Z^{\prime }Z+I\lambda_{1} & Z^{\prime }W & Z^{\prime }V\\ W^{\prime }X & W^{\prime }Z & W^{\prime }W+I\lambda_{2} & W^{\prime }V\\ T^{\prime }X & T^{\prime }Z & T^{\prime }W & T^{\prime }T+I\lambda_{3} \end{array}\right]}^{-1}[\begin{array}{c} X^{\prime }y\\ Z^{\prime }y\\ W^{\prime }y\\ T^{\prime }y \end{array}];$$

where, $\lambda_{1}=\sigma_{e}^{2}/\sigma_{g}^{2}=\frac{1-h^{2}-b^{2}-p^{2}}{h^{2}}$; $\lambda_{2}=\sigma_{e}^{2}/\sigma_{b}^{2}=\frac{1-h^{2}-b^{2}-p^{2}}{b^{2}}$; $\lambda_{3}=\sigma_{e}^{2}/\sigma_{p}^{2}=\frac{1-h^{2}-b^{2}-p^{2}}{p^{2}}.\;h^{2}=\frac{\sigma_{g}^{2}}{\sigma_{g}^{2}+\sigma_{b}^{2}+\sigma_{p}^{2}+\sigma_{e}^{2}}$ is the broad-sense heritability; $b^{2}=\frac{\sigma_{b}^{2}}{\sigma_{g}^{2}+\sigma_{b}^{2}+\sigma_{p}^{2}+\sigma_{e}^{2}}$ is the coefficient of determination for the effects between blocks; and $p^{2}=\frac{\sigma_{p}^{2}}{\sigma_{g}^{2}+\sigma_{b}^{2}+\sigma_{p}^{2}+\sigma_{e}^{2}}$ is the coefficient of determination for permanent environmental effects.

The estimators of the variance components associated with this model using the expectation–maximization algorithm are [33]: $\sigma_{g}^{2}=[{\hat{g}}^{\prime }\hat{g}+\sigma_{e}^{2}\;tr\;(C^{22})]/q;\;\sigma_{b}^{2}=[{\hat{b}}^{\prime }\hat{b}+\sigma_{e}^{2}\;tr\;(C^{33})]/r;\;\sigma_{p}^{2}=[{\hat{p}}^{\prime }\hat{p}+\sigma_{e}^{2}\;tr\;(C^{44})]/N$; and $\sigma_{e}^{2}=[y^{\prime }y-{\hat{u}}^{\prime }X^{\prime }y-\hat{g}Z^{\prime }y-\hat{b}W^{\prime }y-\hat{p}V^{\prime }y]/[N-r(X)]$; where, C is the generalized inverse of the matrix of coefficients of mixed model equations; tr is the trace matrix operator; r(X) is the rank of matrix X (number of linearly independent columns); N-r(X) is the degrees of freedom of the error; N, q, and r are the total number of data, clones, and blocks, respectively.

The coefficient of genetic variation (CV_g_), coefficient of residual variation (CV_e_), and coefficient of relative variation (CV_r_) were calculated, respectively, with the following equations [33]: ${CV}_{g}=(\sigma_{g}^{2}/\mu )x100;\;{CV}_{e}=(\sigma_{e}^{2}/\mu )x100$; and CV_r_ = CV_g_/CV_e_; where μ is the overall average. The genotypic correlation (Pearson’s correlation) among the evaluated traits in the conilon and robusta coffee populations were estimated according to Resende [32]. The classification rules of the parameter estimates (heritability, correlation, accuracy) were based on Resende [36], Resende and Alves [37] and Resende and Duarte [38].

#### Hybrid coffee population

To evaluate the hybrid families, the following linear mixed model was used [32]:

y=Xu+Tc+Wf+Zm+Qs+Sb+e;

where, y is the vector of phenotypic data; u is the vector of the effects of years or harvests (assumed as fixed) added to the overall average; c is the vector of the effects of the specific combining ability between the conilon and robusta genitors (assumed as random); f is the vector of the effects of the general combining ability of the robusta female parent (assumed as random); m is the vector of the general combining ability effects of the conilon male parent (assumed as random); s is the vector of the individual permanent environmental effects (assumed as random); b is the vector of block permanent environmental effects (assumed as random); and e is the vector of residuals (random). The capital letters (X, T, W, Z, Q and S) represent the incidence matrices of these effects.

The average and variance structures associated with this model are [33]: $y|u,\;V∼N(Xu,\;V);\;m|\sigma_{m}^{2}∼N(0,\;I\sigma_{m}^{2});\;f|\sigma_{f}^{2}∼N(0,\;I\sigma_{f}^{2});\;c|\sigma_{c}^{2}∼N(0,\;I\sigma_{c}^{2})$; $s|\sigma_{s}^{2}∼N(0,\;I\sigma_{s}^{2});\;b|\sigma_{b}^{2}∼N(0,\;I\sigma_{b}^{2})$; and $e|\sigma_{e}^{2}∼N(0,\;I\sigma_{e}^{2})$; where, V is the phenotypic variance and equals $ZZ^{\prime }\sigma_{m}^{2}+WW^{\prime }\sigma_{f}^{2}+{TT}^{\prime }\sigma_{c}^{2}+QQ^{\prime }\sigma_{s}^{2}+{SS}^{\prime \sigma_{b}^{2}}+I\sigma_{e}^{2}$; I is an identity matrix; and $\sigma_{m}^{2},\;\sigma_{f}^{2},\;\sigma_{c}^{2},\;\sigma_{s}^{2},\;\sigma_{b}^{2}$ and $\sigma_{e}^{2}$ are the variances among the conilon and robusta genitors, the specific combining ability among the conilon and robusta genitors, the individual permanent environment, the block permanent environment, and the residuals, respectively.

The mixed model equations associated with this model are [33]:

$$[\begin{array}{c} \hat{u}\\ \hat{m}\\ \hat{f}\\ \hat{c}\\ \hat{s}\\ \hat{b} \end{array}]={\left[\begin{array}{cccccc} X^{\prime }X & X^{\prime }Z & X^{\prime }W & X^{\prime }T & X^{\prime }Q & X^{\prime }S\\ Z^{\prime }X & Z^{\prime }Z+I\lambda_{1} & Z^{\prime }W & Z^{\prime }T & Z^{\prime }Q & Z^{\prime }S\\ W^{\prime }X & W^{\prime }Z & W^{\prime }W+I\lambda_{2} & W^{\prime }T & W^{\prime }Q & W^{\prime }S\\ T^{\prime }X & T^{\prime }Z & T^{\prime }W & T^{\prime }T+I\lambda_{3} & T^{\prime }Q & T^{\prime }S\\ Q^{\prime }X & Q^{\prime }Z & Q^{\prime }W & Q^{\prime }T & Q^{\prime }Q+I\lambda_{4} & Q^{\prime }S\\ S^{\prime }X & S^{\prime }Z & S^{\prime }W & S^{\prime }T & S^{\prime }Q & S^{\prime }S+I\lambda_{5} \end{array}\right]}^{-1}[\begin{array}{c} X^{\prime }y\\ Z^{\prime }y\\ W^{\prime }y\\ T^{\prime }y\\ Q^{\prime }y\\ S^{\prime }y \end{array}];$$

where, $\lambda_{1}=\sigma_{e}^{2}/\sigma_{m}^{2}=\frac{1-m^{2}-f^{2}-c^{2}-s^{2}-b^{2}}{m^{2}}$; $\lambda_{2}=\sigma_{e}^{2}/\sigma_{f}^{2}=\frac{1-m^{2}-f^{2}-c^{2}-s^{2}-b^{2}}{f^{2}}$; $\lambda_{3}=\sigma_{e}^{2}/\sigma_{c}^{2}=\frac{1-m^{2}-f^{2}-c^{2}-s^{2}-b^{2}}{c^{2}}$; $\lambda_{4}=\sigma_{e}^{2}/\sigma_{s}^{2}=\frac{1-m^{2}-f^{2}-c^{2}-s^{2}-b^{2}}{s^{2}}$; $\lambda_{5}=\sigma_{e}^{2}/\sigma_{b}^{2}=\frac{1-m^{2}-f^{2}-c^{2}-s^{2}-b^{2}}{b^{2}}.\;m^{2}=\frac{\sigma_{m}^{2}}{\sigma_{m}^{2}+\sigma_{f}^{2}+\sigma_{c}^{2}+\sigma_{s}^{2}+\sigma_{b}^{2}+\sigma_{e}^{2}}$ is the coefficient of determination of the overall combining ability of the conilon parent; $f^{2}=\frac{\sigma_{f}^{2}}{\sigma_{m}^{2}+\sigma_{f}^{2}+\sigma_{c}^{2}+\sigma_{s}^{2}+\sigma_{b}^{2}+\sigma_{e}^{2}}$ is the coefficient of determination of the overall combining ability of the robusta parent; $c^{2}=\frac{\sigma_{g}^{2}}{\sigma_{m}^{2}+\sigma_{f}^{2}+\sigma_{c}^{2}+\sigma_{s}^{2}+\sigma_{b}^{2}+\sigma_{e}^{2}}$ is the coefficient of determination of the specific combining ability between conilon and robusta genitors; $s^{2}=\frac{\sigma_{p}^{2}}{\sigma_{m}^{2}+\sigma_{f}^{2}+\sigma_{c}^{2}+\sigma_{s}^{2}+\sigma_{b}^{2}+\sigma_{e}^{2}}$ is the coefficient of determination of the individual permanent environment; and $b^{2}=\frac{\sigma_{b}^{2}}{\sigma_{m}^{2}+\sigma_{f}^{2}+\sigma_{c}^{2}+\sigma_{s}^{2}+\sigma_{b}^{2}+\sigma_{e}^{2}}$ is the coefficient of determination of block permanent environment.

The estimators of the variance components associated with this model using the expectation-maximization algorithm are [33]: $\sigma_{m}^{2}=[{\hat{m}}^{\prime }\hat{m}+\sigma_{e}^{2}\;tr\;(C^{22})]/q;\;\sigma_{f}^{2}=[{\hat{f}}^{\prime }\hat{f}+\sigma_{e}^{2}\;tr\;(C^{33})]/n;\;\sigma_{c}^{2}=[{\hat{c}}^{\prime }\hat{c}+\sigma_{e}^{2}\;tr\;(C^{44})]/m;\;\sigma_{s}^{2}=[{\hat{s}}^{\prime }\hat{s}+\sigma_{e}^{2}\;tr\;(C^{55})]/N;\;\sigma_{b}^{2}=[{\hat{p}}^{\prime }\hat{p}+\sigma_{e}^{2}\;tr\;(C^{66})]/l$; and $\sigma_{e}^{2}=[y^{\prime }y-{\hat{u}}^{\prime }X^{\prime }y-\hat{m}Z^{\prime }y-\hat{f}W^{\prime }y-\hat{c}T^{\prime }y-\hat{s}Q^{\prime }y-\hat{b}S\prime y]/[N-r(X)]$; where, C is the generalized inverse of the matrix of coefficients of mixed-model equations; tr is the trace matrix operator; r(X) is the rank of matrix X (number of linearly independent columns); N-r(X) is the degrees of freedom of the error; and N, q, n, m, and l are the total number of data, genitors in the conilon group, genitors in the robusta group, combinations between the conilon and robusta groups, and blocks, respectively.

The coefficient of genetic variation (CV_g_), coefficient of residual variation (CV_e_), coefficient of relative variation (CV_r_) and genotypic correlation (Pearson’s correlation) among the evaluated traits in the hybrid coffee population were calculated as described above. The parameter estimates (heritability, correlation, accuracy) were classified as described above.

#### Likelihood ratio test

The significance of the random effects of the statistical models was tested using the likelihood ratio test (LRT) according to the following expression [34]:

$$LRT={-2(LogL-LogL}_{R});$$

where, LogL is the logarithm of the maximum (L) of the restricted likelihood function of the full model; and LogL_R_ is the logarithm of the maximum (L_R_) of the restricted likelihood function of the reduced model (without the effect being tested).

#### Genetic selection

The selection of superior conilon and robusta coffee clones and hybrid families was performed using the Mulamba-Rank index [32]. This index consists of classifying the genotypes in order of performance for each trait then calculating the average rank of each genotype for all categories. Based on the results of this index, the gains from selection were estimated, considering the predicted genotypic values of the selected genotypes.

## Results and discussion

### Conilon coffee population

For all evaluated traits, the genotypic effects were significant (Table 1), indicating the existence of genetic variability among clones. Ramalho et al. [35] also observed genetic variability in their analysis of conilon coffee clones. Except for the trait time of fruit ripening, the effects of block were significant (Table 1). Moreover, except for the traits incidence of cercosporiosis, time of fruit ripening, and fruit size, the effects of the individual permanent environment were significant (Table 1).

**Table 1:** Deviance and likelihood ratio test (LRT) for the following traits: Vegetative vigor (VV), incidence of rust (IR), incidence of cercosporiosis (IC), plant height (PH), canopy diameter (CD), fruit ripening time (FT), fruit size (FS), and yield per plant (YP), evaluated in the conilon coffee population.

	Deviance				LRT		
Trait	CM	GE	BE	PEE	GE	BE	PEE
VV	2878.78	2894.50	2938.41	3002.77	15.72**	59.63**	123.99**
IR	1002.46	1065.39	1014.77	1014.70	62.93**	12.31**	12.24**
IC	-1799.40	-1762.85	-1794.98	-1799.26	36.55**	4.42*	0.14^ns^
PH	13208.80	13214.66	13267.39	13563.83	5.86*	58.59**	355.03**
DC	14101.69	14106.77	14174.04	14258.11	5.08*	72.35**	156.42**
FT	516.76	587.29	519.57	517.36	70.53**	2.81^ns^	0.60^ns^
FS	-367.56	-268.33	-359.93	-365.18	99.23**	7.63**	2.38^ns^
YP	5288.05	5300.48	5298.06	5295.79	12.43**	10.01**	7.74**

CM: complete model; GE: genotypic effect; BE: block effect; PEE: permanent environmental effect.

^ns^, * and **: not significant, significant at 5% and significant at 1%, respectively, based on the Chi-square test with 1 degree of freedom.

Estimates of genetic parameters (heritability, repeatability, genetic correlation) are fundamental for genetic improvement, because they help to define the best breeding and selection strategies for improvement and predict gains from selection [36]. The traits vegetative vigor, rust and cercosporiosis incidence, plant height, canopy diameter, and yield per plant showed individual broad-sense heritability estimates of low magnitude (0.01≤h^2^≤0.15) (Table 2). Meanwhile, fruit ripening time and fruit size showed individual broad-sense heritability estimates of moderate magnitude (0.15<h^2^<0.50) (Table 2).

**Table 2:** Estimates of variance components and genetic and environmental parameters for the following traits: Vegetative vigor (VV), incidence of rust (IR), incidence of cercosporiosis (IC), plant height (PH), canopy diameter (CD), fruit ripening time (FT), fruit size (FS) and yield per plant (YP), evaluated in the conilon coffee population.

Component / Parameter	Trait							
	VV	IR	IC	PH	CD	FT	FS	YP
$\sigma_{g}^{2}$	0.14	0.10	0.01	26.45	21.67	0.10	0.08	1.53
$\sigma_{b}^{2}$	0.20	0.01	0.00	78.75	95.63	0.00	0.00	0.56
$\sigma_{p}^{2}$	0.40	0.04	0.00	225.45	164.34	0.01	0.01	1.77
$\sigma_{e}^{2}$	1.32	0.54	0.11	269.27	485.08	0.49	0.23	22.40
$\sigma_{phen}^{2}$	2.06	0.69	0.13	599.92	766.71	0.60	0.33	26.26
h^2^	0.07	0.14	0.08	0.04	0.03	0.16	0.25	0.06
ρ	0.36	0.21	0.09	0.55	0.37	0.19	0.29	0.15
b^2^	0.10	0.02	0.01	0.13	0.12	0.01	0.01	0.02
p^2^	0.19	0.06	0.01	0.38	0.21	0.02	0.03	0.07
μ	5.79	2.06	2.08	146.64	127.94	1.91	1.98	8.11
CV_g_ (%)	6.52	15.14	4.70	3.51	3.64	16.27	14.43	15.26
CV_e_ (%)	19.86	35.79	16.31	11.19	17.21	36.65	24.33	58.32
CV_r_	0.33	0.42	0.29	0.31	0.21	0.44	0.59	0.26
$\bar{r_{\hat{g}g}}$	0.70	0.86	0.78	0.56	0.53	0.84	0.89	0.67

$\sigma_{g}^{2}$: genotypic variance; $\sigma_{b}^{2}$: environmental variance between blocks; $\sigma_{p}^{2}$: permanent environmental variance; $\sigma_{e}^{2}$: residual variance; $\sigma_{phen}^{2}$: phenotypic variance; h^2^: individual broad-sense heritability; ρ: coefficient of repeatability; b^2^: coefficient of environmental determination between blocks; p^2^: coefficient of determination of permanent environment; μ: overall average; CV_g_ (%): coefficient of genetic variation; CV_e_ (%):coefficient of residual variation; CV_r_: coefficient of relative variation; $\bar{r_{\hat{g}g}}$: average selective accuracy.

The traits rust incidence, cercosporiosis incidence, fruit ripening time, fruit size, and yield per plant presented low estimates for repeatability (*ρ*≤0.30) (Table 2). The traits vegetative vigor, plant height, and canopy diameter presented medium estimates for repeatability (0.30<*ρ*<0.60) (Table 2). The relative coefficients of variation were less than unity for all evaluated traits, given that the largest proportion of phenotypic variation was due to uncontrolled environmental effects (Table 2)

In the context of genetic evaluation, the most important statistical parameter for assessing the experimental quality and predicted genotypic values is the selective accuracy [37]. This parameter reflects the quantity and quality of information and procedures used in the prediction of genetic values and depends on the heritability and repeatability of the analyzed trait [38]. The average accuracies for the traits plant height, canopy diameter, and yield per plant showed moderate magnitude ($0.50\leq \bar{r_{\hat{g}g}}<0.70$) (Table 2). The average accuracies for the traits vegetative vigor, rust incidence, cercosporiosis incidence, fruit ripening time, and fruit size showed high magnitude ($0.70\leq \bar{r_{\hat{g}g}}<0.90$) (Table 2). Similar results were reported by Ramalho et al. [35] in their evaluation of conilon coffee clones.

Genotypic correlation measures the degree of linear association between two traits, resulting from linkage disequilibrium (transient cause) and/or pleiotropy (permanent cause) [36, 39]. The pairs of traits vegetative vigor/canopy diameter and plant height/canopy diameter showed a high magnitude of correlation (0.67 to 1) (Table 3), while the other pairs of traits showed low (0 to 0.33) or medium magnitude (0.34 to 0.66) correlation (Table 3).

**Table 3:** Genotypic correlation among the following traits: Vegetative vigor (VV), incidence of rust (IR), incidence of cercosporiosis (IC), plant height (PH), canopy diameter (CD), fruit ripening time (FT), fruit size (FS), and yield per plant (YP), evaluated in the conilon coffee population.

	IR	IC	PH	CD	FT	FS	YP
VV	-0.38	-0.55	0.58	0.69	-0.04	0.05	0.42
IR		0.57	-0.20	-0.26	-0.32	0.17	-0.20
IC			-0.20	-0.29	-0.12	0.09	-0.57
PH				0.68	-0.23	0.24	0.18
CD					-0.16	0.15	0.38
FT						-0.28	0.05
FS							0.28

By utilizing the Mulamba-Rank index and considering a selection intensity equal to 21% (15 clones), the predicted gain with selection for the genotypic aggregate was equal to 55% (S4 Table in S2 File). This demonstrates the possibility of significant genetic gains with selection in the conilon coffee population. Carias et al. [40] compared the Additive, Multiplicative, and Mulamba-Rank indices in the selection of conilon coffee genotypes and concluded that the Mulamba-Rank index was the most effective. Ramalho et al. [35], evaluating conilon coffee clones for the trait production of processed coffee, and considering a selection intensity of 10%, estimated gains with selection equal to 58%.

### Robusta coffee population

Except for the trait canopy diameter, the genotypic effects were significant (Table 4), indicating the existence of genetic variability among clones. Mistro et al. [20], evaluating conilon coffee progenies for yield per plant, also found genetic variability. Only the traits canopy diameter and fruit ripening time showed significant block effects (Table 4). In addition, the traits vegetative vigor, cercosporiosis incidence, plant height, and canopy diameter showed significant permanent environmental effects (Table 4).

**Table 4:** Deviance and likelihood ratio test (LRT) for the following traits: Vegetative vigor (VV), incidence of rust (IR), incidence of cercosporiosis (IC), plant height (PH), canopy diameter (CD), fruit ripening time (FT), fruit size (FS), and yield per plant (YP), evaluated in the robusta coffee population.

	Deviance				LRT		
Trait	CM	GE	BE	PEE	GE	BE	PEE
VV	1787.14	1792.16	1788.65	1856.04	5.02*	1.51^ns^	68.90**
IR	-1210.90	-1180.72	-1210.88	-1210.93	30.18**	0.02^ns^	-0.03^ns^
IC	-579.57	-558.39	-578.60	-574.85	21.18**	0.97^ns^	4.72*
PH	8605.00	8610.05	8607.32	8723.20	5.05*	2.32^ns^	118.20**
CD	8502.88	8506.16	8507.01	8612.65	3.28^ns^	4.13*	109.77**
FT	103.74	144.45	111.88	103.83	40.71**	8.14**	0.09^ns^
FS	-369.05	-288.77	-369.03	-369.06	80.28**	0.02^ns^	0.01^ns^
YP	2920.41	2926.86	2922.27	2922.38	6.45*	1.86^ns^	1.97^ns^

CM: complete model; GE: genotypic effect; BE: block effect; PEE: permanent environment effect.

^ns^, * and **: not significant, significant at 5%, and significant at 1%, respectively, based on the Chi-square test with 1 degree of freedom.

The traits vegetative vigor, rust incidence, cercosporiosis incidence, plant height, canopy diameter, and yield per plant showed low magnitude estimates of individual broad-sense heritability (0.01≤h^2^≤0.15) (Table 5). For the traits fruit ripening time and fruit size, the individual broad-sense heritability estimates were of moderate magnitude (0.15<h^2^<0.50) (Table 5). Mistro et al. [20], evaluating progenies of conilon coffee regarding yield per plant, obtained a moderate magnitude heritability estimate.

**Table 5:** Estimates of variance components and genetic and environmental parameters for the following traits: Vegetative vigor (VV), incidence of rust (IR), incidence of cercosporiosis (IC), plant height (PH), canopy diameter (CD), fruit ripening time (FT), fruit size (FS), and yield per plant (YP), evaluated in the robusta coffee population.

Component / Parameter	Trait							
	VV	IR	IC	PH	CD	FT	FS	YP
$\sigma_{g}^{2}$	0.11	0.01	0.02	73.88	45.37	0.09	0.07	2.32
$\sigma_{b}^{2}$	0.03	0.00	0.00	21.60	30.92	0.03	0.00	0.70
$\sigma_{p}^{2}$	0.45	0.00	0.01	347.67	286.71	0.01	0.00	2.20
$\sigma_{e}^{2}$	1.46	0.11	0.18	657.64	611.61	0.36	0.17	33.14
$\sigma_{phen}^{2}$	2.05	0.12	0.21	1100.78	974.61	0.48	0.24	38.36
h^2^	0.05	0.07	0.09	0.07	0.05	0.19	0.29	0.06
ρ	0.29	0.08	0.16	0.40	0.37	0.26	0.30	0.14
b^2^	0.01	0.00	0.01	0.02	0.03	0.05	0.00	0.02
p^2^	0.22	0.00	0.06	0.32	0.29	0.01	0.01	0.06
μ	5.75	1.21	2.07	158.46	135.88	2.55	2.52	7.07
CV_g_ (%)	5.72	7.76	6.79	5.42	4.96	11.92	10.52	21.56
CV_e_ (%)	21.02	27.85	20.22	16.18	18.20	23.59	16.27	81.40
CV_r_	0.27	0.28	0.34	0.34	0.27	0.51	0.65	0.26
$\bar{r_{\hat{g}g}}$	0.58	0.75	0.75	0.59	0.53	0.82	0.87	0.59

$\sigma_{g}^{2}$: genotypic variance; $\sigma_{b}^{2}$: environmental variance between blocks; $\sigma_{p}^{2}$: permanent environmental variance; $\sigma_{e}^{2}$: residual variance; $\sigma_{phen}^{2}$: phenotypic variance; h^2^: individual broad-sense heritability; ρ: repeatability coefficient; b^2^: coefficient of environmental determination between blocks; p^2^: coefficient of determination of permanent environmental effects; μ: general average; CV_g_ (%): coefficient of genetic variation; CV_e_ (%): coefficient of residual variation; CV_r_: coefficient of relative variation; $\bar{r_{\hat{g}g}}$: average selective accuracy.

The traits vegetative vigor, rust incidence, cercosporiosis incidence, fruit ripening time, fruit size, and yield per plant showed low repeatability estimates (ρ≤0.30) (Table 5). Meanwhile, plant height and canopy diameter showed medium repeatability estimates (0.30<ρ<0.60) (Table 5). Fonseca et al. [41], evaluating conilon coffee genotypes for the trait bean yield, obtained repeatability coefficient estimates of moderate magnitudes. The relative coefficients of variation were less than unity for all traits, since the largest proportion of phenotypic variation was due to uncontrolled environmental effects (Table 5).

The average accuracies for vegetative vigor, plant height, canopy diameter, and yield per plant presented moderate magnitude ($0.50\leq \bar{r_{\hat{g}g}}<0.70$) (Table 5), while the average accuracies for rust incidence, cercosporiosis incidence, fruit ripening time, and fruit size were high magnitude ($0.70\leq \bar{r_{\hat{g}g}}<0.90$) (Table 5).

The pairs of traits vegetative vigor/canopy diameter and plant height/canopy diameter showed high magnitude correlation (0.67 to 1) (Table 6), while the other pairs of traits showed low (0 to 0.33) or medium magnitude (0.34 to 0.66) correlation (Table 6).

**Table 6:** Genotypic correlation among the following traits: Vegetative vigor (VV), incidence of rust (IR), incidence of cercosporiosis (IC), plant height (PH), canopy diameter (CD), fruit ripening time (FT), fruit size (FS), and yield per plant (YP), evaluated in the robusta coffee population.

	IR	IC	PH	CD	FT	FS	YP
VV	0.02	-0.11	0.63	0.80	-0.16	0.01	0.36
IR		-0.05	-0.08	0.04	-0.20	-0.20	0.24
IC			-0.07	-0.11	-0.06	0.20	0.01
PH				0.68	-0.19	0.04	0.14
CD					-0.25	0.06	0.37
FT						0.19	-0.10
FS							0.00

Using the Mulamba-Rank index and considering a selection intensity equivalent to 27% (15 clones), the predicted gain with selection for the genotypic aggregate was equal to 32% (S5 Table in S2 File), a fact that demonstrates the possibility of significant genetic advancement with selection in the robusta coffee population.

### Hybrid coffee population

The selection of hybrids resulting from crosses between individuals of different varietal groups is particularly relevant for the success of *C*. *canephora* breeding programs [19]. In this study, the effects of specific combining ability were significant for the traits vegetative vigor and plant height (Table 7), indicating the existence of heterosis for these traits. The effects of general combining ability of conilon and robusta parents were significant for the traits rust incidence and fruit ripening time, respectively (Table 7). Permanent environmental effects were significant for the traits vegetative vigor, rust incidence, plant height, canopy diameter, and fruit ripening time (Table 7). The effects of block were significant only for yield per plant (Table 7).

**Table 7:** Deviance and likelihood ratio test (LRT) for the following traits: Vegetative vigor (VV), incidence of rust (IR), plant height (AP), canopy diameter (CD), fruit ripening time (FT), fruit size (FS), and yield per plant (YP), evaluated in the hybrid coffee population.

Trait	Deviance						LRT				
	CM	SCAE	GCAEC	GCAER	PEE	BE	SCAE	GCAEC	GCAER	PEE	BE
VV	1272.16	1278.42	1275.34	1272.15	1444.7	1274.78	6.26*	3.18^ns^	-0.01^ns^	172.54**	2.62^ns^
IR	765.49	766.02	772.23	766.01	819.10	765.62	0.53^ns^	6.74**	0.52^ns^	53.61**	0.13^ns^
PH	8607.12	8615.25	8607.12	8608.74	8748.84	8610.42	8.13**	0.00^ns^	1.62^ns^	141.72**	3.30^ns^
CD	9122.62	9125.39	9123.06	9122.61	9249.89	9123.65	2.77^ns^	0.44^ns^	-0.01^ns^	127.27**	1.03^ns^
FT	557.88	558.56	558.14	564.48	564.20	557.96	0.68^ns^	0.26^ns^	6.60*	6.32*	0.08^ns^
FS	-336.83	-336.83	-336.01	-336.83	-336.82	-333.43	0.00^ns^	0.82^ns^	0.00^ns^	0.01^ns^	3.40^ns^
YP	3480.63	3483.77	3480.63	3480.63	3480.67	3485.53	3.14^ns^	0.00^ns^	0.00^ns^	0.04^ns^	4.90*

CM: complete model; SCAE: specific combining ability effect; GCAEC: general combining ability effect of the conilon parent; GCAER: general combining ability effect of the robusta parent; PEE: permanent environmental effect; EB: block effect.

^ns^, * and **: not significant, significant at 5%, and significant at 1%, respectively, based on the Chi-square test with 1 degree of freedom.

The traits canopy diameter, fruit size, and yield per plant showed individual interpopulation broad-sense heritability estimates of low magnitudes ($0.01\leq h_{g}^{2}\leq 0.15$) (Table 8). For the traits vegetative vigor, rust incidence, plant height, and fruit ripening time the interpopulation individual broad-sense heritability estimates were of moderate magnitude ($0.15<h_{g}^{2}<0.50$) (Table 8). Carvalho et al. [19], evaluating conilon coffee hybrids for the traits rust incidence, plant height, canopy diameter, vegetative vigor, and yield, obtained individual narrow-sense heritability estimates of moderate magnitudes.

**Table 8:** Estimates of variance components and genetic and environmental parameters for the traits: Vegetative vigor (VV), incidence of rust (IR), plant height (PH), canopy diameter (CD), fruit ripening time (FT), fruit size (FS), and yield per plant (YP), evaluated in the hybrid coffee population.

Genetic Parameters	Deviance						
	VV	IR	PH	CD	FT	FS	YP
$\sigma_{m}^{2}$	0.00	0.02	69.72	1.44	0.08	0.00	0.01
$\sigma_{f}^{2}$	0.12	0.07	1.17	19.85	0.01	0.00	0.01
$\sigma_{c}^{2}$	0.09	0.01	67.49	45.08	0.01	0.00	0.90
$\sigma_{s}^{2}$	0.47	0.14	227.91	325.55	0.07	0.00	0.30
$\sigma_{b}^{2}$	0.03	0.00	28.05	17.37	0.00	0.01	1.13
$\sigma_{e}^{2}$	0.80	0.58	466.39	753.13	0.67	0.15	35.97
$\sigma_{phen}^{2}$	1.51	0.83	860.73	1162.43	0.85	0.15	38.32
m^2^	0.00	0.02	0.08	0.00	0.10	0.00	0.00
f^2^	0.08	0.09	0.00	0.02	0.01	0.01	0.00
c^2^	0.06	0.01	0.08	0.04	0.02	0.00	0.02
s^2^	0.31	0.17	0.26	0.28	0.08	0.01	0.01
b^2^	0.02	0.01	0.03	0.01	0.01	0.04	0.03
ρ	0.47	0.29	0.46	0.35	0.21	0.05	0.06
$h_{m}^{2}$	0.00	0.08	0.32	0.00	0.38	0.00	0.00
$h_{f}^{2}$	0.32	0.35	0.01	0.07	0.03	0.03	0.00
$h_{a}^{2}$	0.16	0.21	0.16	0.04	0.21	0.02	0.00
$h_{d}^{2}$	0.23	0.06	0.31	0.16	0.07	0.00	0.09
$h_{g}^{2}$	0.39	0.27	0.48	0.19	0.27	0.02	0.10
μ	6.26	1.45	174.26	158.24	2.02	2.13	8.24
CV_g_ (%)	4.67	7.40	4.71	4.24	5.84	0.25	11.53
CV_e_ (%)	14.30	52.72	12.39	17.34	40.47	17.91	72.78
CV_r_	0.33	0.14	0.38	0.24	0.14	0.01	0.16
$\bar{r_{\hat{g}g}}$	0.60	0.39	0.67	0.57	0.42	0.05	0.55

$\sigma_{m}^{2}$: genetic variance among the genitors of the conilon group; $\sigma_{f}^{2}$: genetic variance among the genitors of the robusta group; $\sigma_{c}^{2}$: variance of the specific combining ability among the conilon and robusta genitors; $\sigma_{s}^{2}$: variance of the individual permanent environmental effects; $\sigma_{b}^{2}$: variance of block permanent environmental effects; $\sigma_{e}^{2}$: residual variance; $\sigma_{phen}^{2}$: phenotypic variance; m^2^: coefficient of determination of the general combining ability of the conilon parent; f^2^: coefficient of determination of the general combining ability of the robusta parent; c^2^: coefficient of determination of the specific combining ability of the conilon and robusta genitors; s^2^: coefficient of determination of the individual permanent environmental effects; b^2^: coefficient of determination of block permanent environmental effects; ρ: coefficient of repeatability, $h_{m}^{2}$: individual narrow-sense heritability in the conilon coffee population; $h_{f}^{2}$: individual narrow-sense heritability in the robusta coffee population; $h_{a}^{2}$: interpopulation individual narrow-sense heritability, average for the two populations; $h_{d}^{2}$: interpopulation individual heritability of dominance effects; $h_{g}^{2}$: interpopulation individual broad-sense heritability; μ: overall average; CV_g_ (%): coefficient of genetic variation; CV_e_ (%): coefficient of residual variation; CV_r_: coefficient of relative variation; $\bar{r_{\hat{g}g}}$: average selective accuracy.

The estimates of individual narrow-sense heritability were greater than 10% for plant height and fruit ripening time in the conilon population, and vegetative vigor and rust incidence in the robusta population (Table 8). The individual heritability estimates for the interpopulation dominance effects were greater than 10% only for the traits vegetative vigor, plant height, and canopy diameter (Table 8), indicating the occurrence of heterosis for these traits.

The traits rust incidence, fruit ripening time, fruit size, and yield per plant presented low repeatability estimates (ρ≤0.30) (Table 8). Meanwhile, the traits vegetative vigor, plant height, and canopy diameter presented medium repeatability estimates (0.30<ρ<0.60) (Table 8). The relative coefficients of variation were less than unity for all evaluated traits, given that the largest proportion of phenotypic variation was due to uncontrolled environmental effects (Table 8).

The average accuracies for the traits rust incidence, fruit ripening time, and fruit size presented low magnitude ($0.01\leq \bar{r_{\hat{g}g}}<0.50$) (Table 8), while the average accuracies for vegetative vigor, plant height, canopy diameter, and yield per plant showed moderate magnitude ($0.50\leq \bar{r_{\hat{g}g}}<0.70$) (Table 8).

The pairs of traits vegetative vigor/plant height, vegetative vigor/canopy diameter, and plant height/canopy diameter showed high magnitude correlation (0.67 to 1) (Table 9), while the other pairs of traits showed low (0 to 0.33) or medium magnitude (0.34 to 0.66) correlation (Table 9).

**Table 9:** Genotypic correlation among the following traits: Vegetative vigor (VV), incidence of rust (IR), plant height (PH), canopy diameter (CD), fruit ripening time (FT), fruit size (FS) and yield per plant (YP), evaluated in the hybrid coffee population.

Trait	IR	PH	CD	FT	FS	YP
VV	0.23	0.82	0.88	-0.15	0.07	0.32
IR		0.29	0.34	-0.50	0.26	0.23
PH			0.86	-0.17	0.29	0.30
CD				-0.21	0.12	0.44
FT					-0.31	-0.01
FS						-0.13

Using the Mulamba-Rank index and considering a selection intensity equal to 50% (10 families), the predicted gains with selection for the genotypic aggregate was equal to 17% (S6 Table in S2 File), demonstrating the possibility of significant genetic gains with selection in the hybrid population.

### Breeding strategies for productivity and disease resistance traits

In *C*. *canephora*, especially in Brazil, there is a lack of comparative studies involving alternative breeding strategies stemming from intrapopulation and interpopulation improvement programs in this species. The objectives of this study were to evaluate parents and families from the populations of conilon, robusta, and its hybrids and to define the best breeding and selection strategies for productivity and disease resistance traits.

*C*. canephora breeding germplasm is based on two contrasting groups native from Africa: the Guinean group (from Occidental Africa) and Congolese group (from Central Africa). In terms of breeding strategies of these species, three main ones can be used: intrapopulation recurrent selection within groups (RS); interpopulation (reciprocal) recurrent selection between groups (RRS); intrapopulation recurrent selection within the hybrid (crossed) population (RSH). RRS and RSH are suitable to improve heterosis. Studies performed in Ivory Coast showed that hybrids between these groups express heterosis [10, 42]. In Brazil, the main representant of the Congolese group is known as conilon and the main representant of the Guinean group is known as robusta. Hybridization between parents of these groups has been performed in Brazil [19], as a mean to get complementary traits and also to exploit heterosis. Then, RRS or RSH strategies can be used. Currently, the RS with cloning of the best clones is the approach more used.

The best breeding strategy can be proposed by looking at the results from the three populations. The main results that can help the breeder are: high or favorable general mean of the population; good (higher than 2/3) selective accuracy in the main traits (this reflects also good genetic control and high genetic variability); favorable (and higher than 1/2) correlation between main traits; significant specific combining ability and high level of dominance, which demonstrate the presence of heterosis.

Significant specific combining ability and high level of dominance were found in the hybrid population for the traits vegetative vigor and plant height (Tables 7 and 8), indicating the existence of heterosis for these traits. Then in such aspect they are suitable for RRS or RSH strategies. Looking at the relevance of these two traits for the breeding we should analyze their correlation with the two principal traits (breeding objectives): coffee yield and rust resistance.

Such correlation are: vegetative vigor with yield per plant (0.42, 0.36 and 0.32, for conilon, robusta and hybrid populations, respectively); vegetative vigor with incidence of rust (-0.38, 0.00 and 0.23, for conilon, robusta and hybrid populations, respectively); plant height with yield per plant (0.18, 0.14 and 0.30, for conilon, robusta and hybrid populations, respectively); plant height with incidence of rust (-0.20, -0.08 and 0.29, for conilon, robusta and hybrid populations, respectively). And other involved traits have correlation: vegetative vigor with plant height (0.58, 0.63 and 0.82, for conilon, robusta and hybrid populations, respectively); incidence of rust with yield per plant (-0.20, 0.24 and 0.23, for conilon, robusta and hybrid populations, respectively).

For conilon it can be seen no meaningful correlation between the traits (vegetative vigor and plant height) subjected to RRS or RSH strategies, with yield per plant and incidence of rust (all correlation below 0.50). The own vegetative vigor and plant height, had correlation of 0.58. These results reveal that the RRS or RSH strategies for vegetative vigor and plant height will not jeopardize the improvement of yield per plant and incidence of rust. However, they will not help either. Yield per plant and incidence of rust shows correlation of -0.20, then allowing the simultaneous improvement of both.

For robusta it can also be seen no meaningful correlation between the traits (vegetative vigor and plant height) subjected to RRS or RSH strategies, with yield per plant and incidence of rust (all correlation below 0.50). These results reveal that the RRS or RSH strategies for vegetative vigor and plant height will not jeopardize the improvement of yield per plant and incidence of rust (correlation of 0.24 between them), whereas they will not help either. For hybrid, the same conclusions can be drawn.

Concerning the selective accuracy for the traits, for conilon, the only below the 0.67 threshold were plant height and canopy diameter. This reveals a very favorable situation for breeding. Concerning the general mean of the population, the values are 8.11 and 2.06 for the breeding objectives yield per plant and incidence of rust. For robusta, the selective accuracy of the traits showed better results for disease resistance and fruit traits. Considering both populations together, only plant height and canopy diameter are at unfavorable situation. Then, it seems, in this aspect, that only vegetative vigor is suitable for SRR. For the heterotic traits vegetative vigor and plant height the general means are 5.79 and 146.64, respectively, for conilon; 5.75 and 158.46, respectively, for robusta; and 6.26 and 158.24, respectively, for the hybrid. All values are similar for the three populations, except by the plant height in robusta, which is higher.

For robusta, the general mean of the population showed the values are 7.07 and 1.25 for the breeding objectives yield per plant and incidence of rust. Then, the conilon showed slightly better for yield per plant and the robusta showed slightly better for incidence of rust. In this way, the selection and breeding in the hybrid population can be recommended as ideal, given the complementary involving yield per plant and incidence of rust. Results for the hybrid population confirm this, as the general mean of the population gave the values are 8.24 and 1.45 for the breeding objectives yield per plant and incidence of rust. We can conclude that the conilon and the hybrid are slightly better than robusta for yield per plant. After the choice to conduct the improvement of the *Coffee canephora* in the hybrid population, a decision should be made between RRS or RSH. As only vegetative vigor is suitable for RRS to increase heterosis and it is not the main trait, we choose the best breeding strategy as RSH in the composed new hybrid population originated from crossing conilon x robusta. This choice allows and enables the simultaneously improvement of all traits of interest. Such strategy is simple, low cost and quicker than the concurrent reciprocal recurrent selection in the two parental populations, and that maximizes the genetic gain for unit of time.

To exploit and improve heterosis in *C*. *canephora*, in Ivory Coast (Africa), Leroy et al. [42] have chosen the RRS, i.e, decided to invest in hybrids. Expressive genetic gain has been reported [10, 43]. Our strategy has also strong features, such as relying on hybrids and being more cost effective and quick.

Concerning the selective accuracy for the traits, for the hybrids the values were below the 0.67 threshold. However, genomic selection can circumvent this by increasing accuracy and speeding the breeding cycle [22, 23, 25].

In conclusion, although the two parental populations themselves have shown low or absent genetic variability, the hybrid population exhibited higher variability and significant specific combining ability for some traits. Then, a sound breeding program can be run for this genetic material. And the best choice of breeding strategy would be the RSH because it is genetically efficient as it keeps and increases the heterosis in the population, is simple, low cost and quicker than RRS, and this maximizes the genetic gain for unit of time.

## Supporting information

S1 File Data files of the conilon, robusta and hybrid coffee populations. (RAR)

S2 File (DOCX)

S1 Dataset (RAR)
