# Supplementary material for: Designing the best breeding strategy for Coffea canephora: Genetic evaluation of pure and hybrid individuals aiming to select for productivity and disease resistance traits
Source: PLoS One. 2021 Dec 29;16(12):e0260997. doi: 10.1371/journal.pone.0260997 (PMC8716045; doi:10.1371/journal.pone.0260997)
Supplement: S2 File — (DOCX) [file pone.0260997.s002.docx]

**Designing the best breeding strategy for *Coffea canephora*: genetic evaluation of pure and hybrid individuals aiming to select for productivity and disease resistance traits**

Emilly Ruas Alkimim^1^, Eveline Teixeira Caixeta^2^, Tiago Vieira Sousa^3^, Itamara Bomfim Gois^4^, Felipe Lopes da Silva^4^, Ney Sussumu Sakiyama^4^, Laércio Zambolim^4^, Rodrigo Silva Alves^5^, Marcos Deon Vilela de Resende^2^

^1^ Federal University of Triângulo Mineiro, Iturama, MG, Brazil.

^2^ Brazilian Agricultural Research Corporation - Embrapa Café, Viçosa, MG, Brazil.

^3^ Federal Institute of Triângulo Mineiro, Campina Verde, MG, Brazil.

^4^ Federal University of Viçosa, Viçosa, MG, Brazil.

^5^ National Institute of Coffee Science and Technology - INCT Café, Lavras, MG, Brazil.

**Corresponding authors:** eveline.caixeta@embrapa.br and marcos.resende@embrapa.br

**Table S1.** Conilon coffee clones.

| UFV 3627-8 | UFV 3628-5 | UFV 3628-32 | UFV 3628-46 | UFV 3629-12 | UFV 3629-30 |
| --- | --- | --- | --- | --- | --- |
| UFV 3627-20 | UFV 3628-10 | UFV 3628-33 | UFV 3628-47 | UFV 3629-15 | UFV 3629-31 |
| UFV 3627-24 | UFV 3628-16 | UFV 3628-35 | UFV 3628-48 | UFV 3629-16 | UFV 3629-32 |
| UFV 3627-25 | UFV 3628-17 | UFV 3628-36 | UFV 3628-51 | UFV 3629-17 | UFV 3629-34 |
| UFV 3627-27 | UFV 3628-20 | UFV 3628-37 | UFV 3628-52 | UFV 3629-20 | UFV 3629-36 |
| UFV 3627-29 | UFV 3628-22 | UFV 3628-38 | UFV 3629-1 | UFV 3629-23 | UFV 3629-37 |
| UFV 3627-30 | UFV 3628-23 | UFV 3628-39 | UFV 3629-4 | UFV 3629-24 | UFV 3629-38 |
| UFV 3627-31 | UFV 3628-24 | UFV 3628-40 | UFV 3629-7 | UFV 3629-25 | UFV 3629-39 |
| UFV 3628-1 | UFV 3628-26 | UFV 3628-42 | UFV 3629-8 | UFV 3629-26 | UFV 3629-? |
| UFV 3628-2 | UFV 3628-27 | UFV 3628-43 | UFV 3629-9 | UFV 3629-27 | UFV 3638-49 |
| UFV 3628-3 | UFV 3628-28 | UFV 3628-44 | UFV 3629-10 | UFV 3629-28 | UFV 513 |

**Table S2.** Robusta coffee clones.

| UFV 3356-71 | UFV 3363-125 | UFV 3370-47 | UFV 3376-9 | UFV Apoatã-1 |
| --- | --- | --- | --- | --- |
| UFV 3356-74 | UFV 3365-144 | UFV 3370-49 | UFV 3377-12 | UFV Apoatã-2 |
| UFV 3356-76 | UFV 3366-134 | UFV 3370-50 | UFV 3630-10 | UFV Apoatã-3 |
| UFV 3357-91 | UFV 3366-138 | UFV 3371-19 | UFV 3630-11 | UFV Apoatã-4 |
| UFV 3357-93 | UFV 3366-139 | UFV 3371-20 | UFV 3630-2 | UFV Apoatã-5 |
| UFV 3358-88 | UFV 3367-101 | UFV 3371-22 | UFV 3630-5 | UFV Apoatã-6 |
| UFV 3360-169 | UFV 3367-105 | UFV 3373-36 | UFV 3630-6 | UFV Apoatã-8 |
| UFV 3360-171 | UFV 3367-96 | UFV 3373-43 | UFV 3630-7 | UFV 514 |
| UFV 3361-148 | UFV 3367-97 | UFV 3374-29 | UFV 3631-10 |  |
| UFV 3361-151 | UFV 3367-98 | UFV 3375-65 | UFV 3631-11 |  |
| UFV 3362-118 | UFV 3368-52 | UFV 3375-66 | UFV 3631-13 |  |
| UFV 3363-122 | UFV 3368-58 | UFV 3376-8 | UFV 3631-9 |  |

**Table S3.** Hybrid coffee families.

| Family | Cross |
| --- | --- |
| H092 | UFV 3366-139 × UFV 513 |
| H093 | UFV 3366-139 × UFV 3629-11 |
| H094 | UFV 3367-98 × UFV 513 |
| H095 | UFV 3366-139 × UFV 3627-31 |
| H097 | UFV 3374-28 × UFV 3627-31 |
| H098 | UFV 3365-144 × UFV 3629-11 |
| H099 | UFV 3365-144 × UFV 513 |
| H0910 | UFV 3367-98 × UFV 3629-11 |
| H0911 | UFV 3367-98 × UFV 3628-2 |
| H0912 | UFV 3366-139 × UFV 3628-2 |
| H0913 | UFV 3365-144 × UFV 3628-2 |
| H0914 | UFV 3374-28 × UFV 513 |
| H0915 | UFV 3373-36 × UFV 3628-2 |
| H0916 | UFV 3367-98 × UFV 3629-25 |
| H0917 | UFV 3365-144 × UFV 3629-25 |
| H0918 | UFV 3373-36 × UFV 513 |
| H0919 | UFV 3373-36 × UFV 3629-11 |
| H0920 | UFV 3373-36 × UFV 3629-25 |
| H0921 | UFV 3374-28 × UFV 3629-11 |
| H0922 | UFV 3373-36 × UFV 3627-31 |

**Table S4.** Classification of the 15 conilon clones selected based on the Mulamba-Rank index considering the traits: vegetative vigor (VV), incidence of rust (IR), incidence of cercospora (IC), plant height (PH), canopy diameter (CD), fruit ripening time (FT), fruit size (FS), and yield per plant (YP).

| Rank | Clone | VV | IR | IC | PH | CD | FT | FS | YP | Mulamba-Rank | Gain (%) |
| --- | --- | --- | --- | --- | --- | --- | --- | --- | --- | --- | --- |
| 1 | UFV 3628-2 | 3 | 3 | 3 | 71 | 1 | 2 | 32 | 7 | 15.25 | 136.07 |
| 2 | UFV 3628-45 | 1 | 1 | 4 | 69 | 4 | 4 | 58 | 16 | 19.63 | 106.45 |
| 3 | UFV 3627-27 | 9 | 14 | 5 | 43 | 17 | 18 | 41 | 13 | 20.00 | 96.81 |
| 4 | UFV 3627-29 | 5 | 6 | 1 | 50 | 10 | 7 | 71 | 15 | 20.63 | 90.73 |
| 5 | UFV 3629-26 | 24 | 22 | 12 | 42 | 15 | 47 | 13 | 6 | 22.63 | 83.44 |
| 6 | UFV 3629-4 | 13 | 5 | 13 | 21 | 29 | 63 | 16 | 22 | 22.75 | 78.70 |
| 7 | UFV 3628-22 | 8 | 52 | 39 | 66 | 7 | 5 | 2 | 12 | 23.88 | 74.09 |
| 8 | UFV 3628-4 | 37 | 12 | 6 | 67 | 11 | 17 | 19 | 25 | 24.25 | 70.41 |
| 9 | UFV 3629-34 | 2 | 9 | 23 | 64 | 3 | 54 | 37 | 5 | 24.63 | 67.33 |
| 10 | UFV 3628-26 | 11 | 37 | 33 | 38 | 28 | 27 | 6 | 19 | 24.88 | 64.76 |
| 11 | UFV 3628-28 | 38 | 59 | 49 | 11 | 2 | 12 | 3 | 26 | 25.00 | 62.63 |
| 12 | UFV 3629-29 | 27 | 18 | 21 | 47 | 13 | 59 | 10 | 14 | 26.13 | 60.22 |
| 13 | UFV 3629-27 | 33 | 15 | 11 | 24 | 35 | 65 | 23 | 4 | 26.25 | 58.17 |
| 14 | UFV 3628-40 | 18 | 30 | 8 | 26 | 41 | 62 | 8 | 18 | 26.38 | 56.40 |
| 15 | UFV 3628-46 | 6 | 32 | 16 | 23 | 9 | 64 | 35 | 27 | 26.50 | 54.84 |

**Table S5.** Ranking of the 15 robusta clones selected based on the Mulamba-Rank index considering the traits: vegetative vigor (VV), incidence of rust (IR), incidence of cercospora (IC), plant height (PH), fruit ripening time (FT), fruit size (FS), and yield per plant (YP).

| Rank | Clone | VV | IR | IC | PH | FT | FS | YP | Mulamba-Rank | Gain (%) | |
| --- | --- | --- | --- | --- | --- | --- | --- | --- | --- | --- | --- |
| 1 | UFV 3631-10 | 18 | 8 | 17 | 7 | 13 | 47 | 13 | 17.57 | 62.20 |  |
| 2 | UFV 3371-20 | 6 | 29 | 28 | 40 | 6 | 8 | 6 | 17.57 | 62.20 |  |
| 3 | UFV Apoatã-2 | 1 | 1 | 4 | 54 | 32 | 2 | 32 | 18.00 | 60.89 |  |
| 4 | UFV 3370-50 | 25 | 2 | 13 | 31 | 46 | 7 | 5 | 18.43 | 59.28 |  |
| 5 | UFV 3357-91 | 44 | 4 | 9 | 12 | 34 | 35 | 3 | 20.14 | 55.37 |  |
| 6 | UFV 3630-5 | 2 | 3 | 1 | 49 | 43 | 41 | 2 | 20.14 | 52.87 |  |
| 7 | UFV 3630-6 | 7 | 28 | 16 | 41 | 24 | 3 | 31 | 21.43 | 49.68 |  |
| 8 | UFV 3356-74 | 17 | 5 | 3 | 21 | 40 | 48 | 17 | 21.57 | 47.23 |  |
| 9 | UFV 3368-52 | 23 | 22 | 34 | 9 | 26 | 37 | 9 | 22.86 | 44.33 |  |
| 10 | UFV 3365-144 | 42 | 10 | 45 | 4 | 28 | 11 | 20 | 22.86 | 42.09 |  |
| 11 | UFV Apoatã-1 | 15 | 6 | 7 | 53 | 20 | 30 | 35 | 23.71 | 39.78 |  |
| 12 | UFV 3360-169 | 10 | 56 | 5 | 43 | 3 | 50 | 4 | 24.43 | 37.51 |  |
| 13 | UFV 3368-58 | 11 | 51 | 56 | 44 | 5 | 6 | 1 | 24.86 | 35.43 |  |
| 14 | UFV 3373-36 | 9 | 12 | 35 | 56 | 4 | 43 | 16 | 25.00 | 33.64 |  |
| 15 | UFV 3358-88 | 26 | 31 | 24 | 2 | 36 | 29 | 30 | 25.43 | 31.94 |  |

The canopy diameter trait was not included in this analysis, as it had non-significant genetic effects (Table 4).

**Table S6.** Ranking of the 10 hybrid families selected based on the Mulamba-Rank index considering the traits: vegetative vigor (VV), incidence of rust (IR), plant height (PH), and fruit ripening time (FT).

| Rank | Hybrid | VV | IR | PH | FT | Mulamba-Rank | Gain (%) |
| --- | --- | --- | --- | --- | --- | --- | --- |
| 1 | H0921 | 2 | 8 | 17 | 3 | 7.5 | 40.0 |
| 2 | H0912 | 9 | 1 | 8 | 13 | 7.8 | 37.7 |
| 3 | H0918 | 12 | 7 | 9 | 5 | 8.3 | 34.0 |
| 4 | H097 | 14 | 2 | 1 | 19 | 9.0 | 29.2 |
| 5 | H0917 | 18 | 5 | 3 | 10 | 9.0 | 26.5 |
| 6 | H099 | 4 | 4 | 12 | 17 | 9.3 | 24.1 |
| 7 | H0919 | 16 | 6 | 2 | 14 | 9.5 | 22.0 |
| 8 | H0913 | 15 | 16 | 7 | 1 | 9.8 | 20.0 |
| 9 | H095 | 3 | 20 | 14 | 2 | 9.8 | 18.5 |
| 10 | H0922 | 1 | 15 | 20 | 4 | 10.0 | 17.0 |

The canopy diameter, fruit size and production per plant traits were not included in this analysis, as they presented non-significant genetic effects (Table 7).
